# The effects of different physiologic concentrations of prolactin in association with reproductive hormones on the incidence of type 2 diabetes mellitus in men: Tehran Lipid and Glucose Study

**DOI:** 10.1186/s12902-022-01225-x

**Published:** 2022-12-05

**Authors:** Atrin Niknam, Fatemeh Mahboobifard, Maryam Rahmati, Faezeh Firouzi, Ehsan Rojhani, Fereidoun Azizi, Fahimeh Ramezani Tehrani

**Affiliations:** 1 Reproductive Endocrinology Research Center, Research Institute for Endocrine Sciences, Shahid Beheshti University of Medical Sciences, Velenjak, Tehran, Iran; 2 Department of Pharmacology, School of Medicine, Shahid Beheshti University of Medical Sciences, Tehran, Iran; 3 Pathology Department of Shahid Beheshti University of Medical Sciences, Tehran, Iran; 4 Endocrine Research Center, Research Institute for Endocrine Sciences, Shahid Beheshti University of Medical Sciences, Tehran, Iran

**Keywords:** Type 2 diabetes mellitus, Prolactin, Physiologic range, Population-based cohort study

## Abstract

**Background:**

Data is inconsistent and, for the most part, not sufficient to demonstrate the association between serum Prolactin (PRL) concentration within the physiologic range and the incidence rate of type 2 Diabetes Mellitus (DM) among men. Moreover, since both PRL and type 2 DM are associated with reproductive hormones, investigating these hormones might improve our understanding of how PRL might impose its effect on the incidence rate of type 2 DM.

**Methods:**

For the present study, 652 eligible men aged 29–70 with a normal baseline PRL concentration were selected from the Tehran Lipid and Glucose Study (TLGS). Participants were sub-classified into three groups (tertiles) according to the serum concentration of PRL and were followed for 15.8 years. The incidence of type 2 DM and PRL, LH, FSH, testosterone, and AMH concentrations were measured. The effect of hormonal variables on the incidence of type 2 DM was estimated using the log-binomial model, adjusted for major confounding factors. The correlations between PRL and the indicators of glucose and lipid metabolism and other hormonal variables were also explored.

**Results:**

In the unadjusted model, PRL was not significantly associated with the incidence rate of type 2 DM (RR = 0.98, 95% CI: 0.94 − 1.03). After adjusting for potential confounders, the inverse effect of AMH on the incidence rate of type 2 DM was the only significant association. The analyses also indicated a significant positive association between PRL and LH/FSH ratio (*r* = 0.1, *P* = 0.01).

**Conclusion:**

No significant association was found between serum PRL concentrations within the physiologic range and the incidence rate of type 2 diabetes mellitus among middle-aged men. Men with higher concentrations of PRL within the physiologic range tended to show higher levels of LH and LH/FSH. AMH was the only variable significantly linked to the incidence rate of type 2 DM in men.

## Background

Prolactin (PRL) is a polypeptide pituitary hormone primarily known for its lactogenic role in mammals [1, 2]. PRL receptors are expressed in multiple organs and are involved in many physiologic functions, including osmoregulation, immune response, growth and development, brain function, reproduction, and metabolism [2–4]. According to preclinical studies, it is evident that serum PRL concentrations within the physiologic range contribute to maintaining normal glucose homeostasis in both sexes [5–7]. To elucidate, PRL enhances the proliferation of pancreatic beta cells, increases glucose-dependent insulin secretion, and prevents pancreatic islet apoptosis. As a result, serum PRL concentrations within the physiologic range might reduce the risk of developing type 2 Diabetes Mellitus (DM) [5–7]. In this context, it has been shown that higher than-normal serum concentrations of PRL (hyperprolactinemia) lead to insulin resistance and glucose intolerance [8, 9]. In addition, the symptomatic state of excess PRL that could be developed via hypothalamic-pituitary axis disorders or drugs -referred to as pathologic hyperprolactinemia- increases post-prandial insulin resistance. This process leads to increased gluconeogenesis together with lipolysis and impairment of peripheral glucose uptake [8–11]. Furthermore, hyperprolactinemia results in abnormal fatty acid metabolism and an aggravated inflammatory state, leading to increased food intake, weight gain, and insulin resistance [12–15]. Therefore, hyperprolactinemia (pathologic or not) might increase the risk of type 2 DM [9, 16]. As a result, PRL has contradictory effects on metabolism; while its concentrations within the physiologic range seem metabolically beneficial, its pathological concentrations have a detrimental effect on metabolism.

Several studies reported a significant inverse association between serum PRL concentration within the physiologic range and the risk of type 2 DM [17–21]. Nonetheless, the literature is inconsistent concerning the association between serum PRL concentrations within the physiologic range and the risk of type 2 DM among men, pointed out in a recent meta-analysis [22]. Although cross-sectional studies have reported a significant association between higher quartiles of serum PRL concentration within the physiologic range and lower risk of type 2 DM in both sexes [19, 20, 23], and large-scale longitudinal studies have demonstrated this association among women [17, 18], data is inconsistent and for the most part not sufficient to establish this association among men [18, 22, 23]. Another possible scenario contributing to this debate is that PRL concentrations within the physiologic range might have a sex-specific effect on glucose metabolism [24, 25]. On the one hand, previous research suggests an association between the risk of type 2 DM and reproductive hormones such as LH, FSH, testosterone, and AMH [26–29]. On the other hand, PRL appears to play a sex-specific role in reproduction and fertility alongside these hormones [30]. Thus, investigating the relationships between PRL and other reproductive hormones might help to explain why men and women have different interactions between PRL and type 2 DM. The paucity of studies properly equipped to answer these questions in the population of men prompted us to conduct a study on a dataset from a large long-term community-based cohort, the Tehran Lipid and Glucose Study (TLGS), to determine whether various concentrations of serum PRL within the physiologic range affect the incidence rate of type 2 DM among men, and to investigate the possibility of involvement of other reproductive hormones in the mechanisms at work.

## Methods

### Study population

The sample for the present study was selected from the male participants of the Tehran Lipid and Glucose Study (TLGS). TLGS is an ongoing large-scale community-based multi-center cohort study initiated in the late 1990s to assess the incidence, prevalence, and risk factors of non-communicable diseases (NCDs) and metabolic disturbances among 3–69 years old residents of a district in Tehran, Iran, using a multistage stratified cluster random sampling technique. From the start, the participants have been followed up in visits three years apart, undergoing complete medical history and anthropometric data acquisition, blood sampling for biochemical tests of serum glucose and lipids, and outcome measurements. Further details of TLGS have been published elsewhere [31].

### Selection criteria

For the present study, we selected all men aged 29–70 who participated in phase 1 of TLGS and had their serum PRL concentration measured. At baseline, all men who had a previous diagnosis of diabetes mellitus (*n* = 99) or serum PRL concentration of more than 20 ng/ml (*n* = 50) were excluded from the study, as well as participants who failed to attend any follow-up sessions (*n* = 27). Any participant taking medications known to elevate serum PRL concentration, such as first and second-generation antipsychotics, cyclic antidepressants, antihypertensives, antihistamines, and antiemetics like Metoclopramide, anyone with persistent headaches that could be suspicious of pituitary adenoma or hypothalamus tumor, cases of chronic renal failure or macroprolactinemia, cases of chest-wall injuries, those taking medications known to alter metabolic parameters, such as insulin, sulfonylurea and thiazolidinediones, beta-blockers and calcium-channel blockers, psychotropic and anti-seizure drugs, lipid-lowering drugs and corticosteroids were all excluded, which left us with a total of 652 eligible healthy men.

The protocol of the present study was designed according to the principles of the Helsinki declaration and was approved by the Ethics committee of the Research Institute for Endocrine Sciences (IR.SBMU.ENDOCRINE.REC.1400.082). Informed written consent was obtained from each participant after providing them with complete descriptions of the study. STROBE reporting guidelines for observational studies were used to design and report the present study.

### Clinical, anthropometric, and laboratory measurements

Details of the measurements have been previously published [31]. Briefly, at baseline and each follow-up session, participants filled out a standard questionnaire regarding their complete medical and family history with the help of two trained physicians. Moreover, they underwent a brief physical examination, including a measurement of their anthropometrics such as weight, standing height, waist circumference (WC), hip circumference (HC), and wrist circumference (WrC). Body mass index (BMI) was calculated by dividing weight (kg) by height squared (m^2^), and waist-to-hip ratio (WHR) was calculated by dividing WC (cm) by HC (cm).

Venous blood samples were taken without excessive venepuncture stress from participants in the morning after 12–14 h of overnight fasting and 2–3 h after waking up, centrifuged for 30–45 min, and stored in -80 °C ultra-freezers until further testing. Fasting plasma glucose (FPG) was measured with the Glucose oxidase technique (Glucose kit, Pars Azmun, Tehran, Iran) in enzymatic colorimetric method, with inter- and intra-assay coefficients of variations (CV) of 2.2% both. A 2-h postprandial glucose test with 75 g glucose was administered for participants who did not use any glucose-lowering medications. Total cholesterol (TC), triglyceride (TG), and high-density lipoprotein (HDL) cholesterol concentrations were measured using an enzymatic colorimetric test (Pars Azmun kit, Tehran, Iran). Low-density lipoprotein (LDL) cholesterol was calculated using the Friedwald formula [32]. Measurement of luteinizing hormone (LH), follicle-stimulating hormone (FSH), and PRL were based on Immunoradiometric assay (IRMA) using a gamma counter (Izotop, Budapest, Hungary, gamma counter: Dream Gamma- 10, Goyang-si, Gyeonggi-do, South Korea). The intra- and inter-assay coefficients of variations (CVs) for LH, FSH and PRL were 2.9% and 3.0%, 1.3% and 1.4%, 2.5% and 2.6%, respectively, at the detection limit of 0.02 mIU/mL, 0.08 mIU/mL and 0.04 ng/mL, respectively. Total AMH concentration was assayed using enzyme immunoassay (EIA) (AMH Gen Π, Beckman Coulter, Inc. Ca, USA, Sunrise, Tecan Co. Salzburg, Austria), with intra- and inter-assay CVs of 3.1% and 3.2%, respectively, at the detection limit of 0.08 ng/ml. Total testosterone concentration was measured using EIA (DRG Instrument, Sunrise, Tecan Co. Salzburg, Austria, GmbH, Germany), with intra- and inter-assay CVs of 5.7% and 8.4%, respectively, at the detection limit of 0.002 ng/ml. All measurements were carried out at the laboratory of the Research Institute for Endocrine Sciences (RIES).

### Definitions

The normal range of serum PRL concentration in an adult male is 3 to 13 ng/ml, which is lower than an adult female [33]. However, since various methods and kits are used to measure PRL, there is some variation in the ranges reported by different laboratories. Based on the detection methods used in our laboratories, the normal/physiologic range of serum PRL concentration was set from 3 to 20 ng/ml. Hyperprolactinemia is a condition where serum PRL concentration exceeds the upper limit of the normal range [34].

Type 2 diabetes mellitus (DM) was diagnosed in the participants who met at least one of the criteria of the American Diabetes Association’s definition of diabetes [35]: 2-h PCPG ≥ 11.1 mmol/L OR FPG ≥ 7 mmol/L OR using any anti-diabetic medication. Also, those with FPG < 5.05 mmol/L and missing data on the 2-hpp glucose test at follow-up were defined as non-diabetic [36]. In addition, they did not have any early symptoms of insulin deficiency, autoimmune-related disease, or any history of type 1 DM in a first-degree relative. Family history of type 2 DM was defined as having at least one first-degree relative diagnosed with type 2 DM.

In our dataset, physical activity was measured using the Modifiable Activity Questionnaire (MAQ), including leisure time, job, and household activities. Appropriate physical activity was defined as more than 600 min of moderate physical activity within a week.

### Statistical analysis

The present study has a power of 80% to detect 0.45% risk reduction in type 2 DM outcome, with a two-sided 5% significance level, and a sample size of 652 male participants. Continuous variables were checked for normal distribution using the one-sample Kolmogorov-Smirnoff test. Categorical variables were expressed as percentages, whereas continuous variables with normal distribution were expressed as mean ± standard deviation (SD), and non-normal distributed variables were expressed as median with interquartile range (IQR 25%– 75%). Participants were sub-classified into three groups according to the serum concentration of PRL. Characteristics of the participants at baseline and last follow-up were compared between the tertiles of PRL, by applying the ANOVA or chi-square test for continuous and categorical data, respectively. Last follow-up was considered a visit at which participants experienced either a DM outcome or the last follow-up in which they have been censored. Bonferroni post-hoc test after ANOVA was applied when an overall statistically significant difference in group means was observed. The Kruskal–Wallis test was applied to compare variables with skewed distribution. After a significant Kruskal–Wallis, Dunn test was used in order to make pairwise comparisons. Furthermore, the incidence rate ratio (IRR) of DM per 1000 persons as well as 95% confidence intervals were estimated in each tertile of PRL.

A scatter plot matrix was drawn, and Spearman’s correlation test was used to explore the correlation between PRL and LH, FSH, LH/FSH, testosterone, and AMH, as well as indicators of glucose and lipid metabolism at the baseline of the study. The effect of hormonal variables on binary type 2 DM outcome was estimated using the log-binomial model, which is a useful approach for computing relative risks [37]. Both unadjusted and adjusted models were fitted, and effect measures (relative risks [RRs]) were calculated with 95% confidence intervals. Based on the literature, potential confounding variables were considered to be age, BMI, smoking status, and family history of DM [22]. Moreover, to check if the effect of PRL on DM depends on the value of hormonal variables, an interaction term between PRL and each hormonal variable was added to the log-binomial model. The Cox regression model was applied to explore the effect of PRL on the age at the onset of diabetes, and hazard ratios were estimated. Statistical analyses were performed using the STATA software package (version 13; STATA Inc., College Station, TX, USA). The significance level was set at *P* < 0.05 and the confidence interval at 95%.

## Results

The characteristics of the study participants at baseline and last follow-up according to their serum concentration of PRL are presented in Table 1. The mean follow-up was 15.8 years (IQR: 13.6 − 17.2). Of all participants, 112 men (17%) were diagnosed with type 2 DM in the course of the study. Age at DM onset was 58 (51 − 67), 56 (51 − 69), and 53.5 (47 − 63) years in the first, second and third PRL tertile, respectively (*P* = 0.2).

**Table 1:** Characteristics of the study participants according to the tertiles of serum concentration of prolactin at baseline and last follow-up

Variables	Serum PRL concentration						Comparison	
	Baseline			Last follow-up^*^			*P*-value^¥^	*P*-value^£^
	Tertile 1 (*N* = 223)	Tertile 2 (*N* = 222)	Tertile 3 (*N* = 207)	Tertile 1 (*N* = 223)	Tertile 2 (*N* = 222)	Tertile 3 (*N* = 207)		
Age (year)^a^	44.3 (10.4)	44.0 (11.3)	43.4 (12)	59.5 (10)	59.5 (10.8)	58.9 (11.4)	0.7	0.8
BMI (kg/m^2^)^a^	25.4 (3.7)	26.0 (4.1)	26 (4.2)	27.2 (4.2)	27.4 (4.6)	28.0 (4.5)	0.2	0.1
Marital status (married), n (%)	213 (95.5)	213 (95.9)	192 (92.7)	210 (94.2)	212 (95.5)	200 (96.6)	0.3	0.5
Education (upper diploma), n (%)	132 (59.2)	117 (52.7)	116 (56)	150 (67.3)	117 (52.7)	128 (61.8)	0.4	0.1
Family history of diabetes, n (%)	56 (25.1)	60 (27.0)	62 (30)	56 (25.1)	60 (27.0)	62 (30)	0.5	0.5
Smoking status (ever), n (%)	112 (50.2)	91 (41.0)	84 (40.6)	119 (53.4)	95 (42.8)	89 (43.0)	0.07	0.3
Physical activity (appropriate), n (%)	44 (19.7)	52 (23.4)	35 (16.9)	95 (42.8)	97 (43.7)	82 (39.6)	0.2	0.7
WC (cm)^a^	87.5 (10)	88.3 (11.5)	88.9 (11)	96.6 (10.8)	96.9 (11.4)	98.2 (11.1)	0.4	0.3
HC (cm)^a^	95.5 (6.6)	96.9 (7.1)	96.6 (7.8)	97.9 (7.4)	98.8 (7.9)	99.4 (8.5)	0.1	0.1
WHR^a^	0.91 (0.06)	0.91 (0.07)	0.92 (0.06)	0.98 (0.06)	0.98 (0.05)	0.99 (0.05)	0.3	0.3
WrC (cm)^a^	17.6 (0.9)	17.7 (0.9)	17.8 (1)	17.7 (1.0)	17.8 (1.0)	17.9 (1.1)	0.6	0.3
SBP (mmHG)^a^	118.7 (19.2)	118.8 (19.4)	119.9 (16)	122.7 (20.9)	122.9 (19.0)	121.9 (15.6)	0.8	0.9
DBP (mmHG)^a^	78.2 (11.2)	76.9 (9.8)	78.5 (11)	79.4 (11.2)	78.6 (10.2)	79.8 (10.8)	0.2	0.5
Total cholesterol (mg/dl)^a^	205.9 (35.6)	205.2 (40.2)	210 (38)	190.9 (37)	188.3 (36.3)	195.4 (42.0)	0.4	0.1
TG (mg/dl)^b^	149 (103 − 203)	157 (109 − 231)	154 (111 − 207)	135 (97 − 185)	138.5 (103 − 198)	138 (99 − 196)	0.3	0.5
LDL (mg/dl)^a^	132.5 (30.1)	130.9 (35.8)	136.5 (34.8)	117.7 (31.6)	113.5 (31)	121.1 (36)	0.2	0.06
HDL (mg/dl)^a^	39.7 (10.5)	38.2 (9.2)	38.7 (9.3)	43.6 (10.4)	42.8 (10.4)	43.2 (9.8)	0.2	0.7
FPG (mg/dl)^a^	90.6 (9.3)	90.0 (9.6)	90.9 (8.5)	101.7 (25)	101.2 (22)	101.6 (22.5)	0.6	0.9
2-h OGTT glucose (mg/dl)^a^	100.3 (29.2)	96.7 (29.7)	96.7 (28)	130.8 (57.8)	133.3 (58.4)	130 (51.9)	0.2	0.8
LH (IU/l)^b^	3.9 (2.7 − 5.5)^**13**^	4.0 (3.0 − 5.1)^**23**^	4.5 (3.5 − 5.8)	-	-	-	**0.003**	**-**
FSH (IU/l)^b^	5.4 (3.9 − 7.8)	5.1 (3.5 − 7.8)	5.1 (3.4 − 8)	-	-	-	0.9	-
LH/FSH ratio^b^	0.75 (0.52 − 1)^**13**^	0.75 (0.53 − 0.97)^**23**^	0.77 (0.59 − 1.2)	-	-	-	**0.04**	**-**
AMH (ng/dl) ^b^	5.7 (3.5 − 8.3)	6.25 (3.6 − 9.4)	6 (3.5 − 8.6)	-	-	-	0.4	-
Testosterone (ng/dl)^b^	4.7 (3.8 − 5.5)	4.9 (4.1 − 5.6)	4.8 (4 − 5.6)	-	-	-	0.3	-
DM, n (%)^†^	-	-	-	35 (15.7)	43 (19.4)	30 (14.5)	-	0.4
Age at DM onset (year)^b^	-	-	-	58 (51 − 67)	56 (51 − 69)	53.5 (47 − 63)	-	0.2

*Abbreviations*: *PRL* Prolactin, *BMI* Body mass index, *WC* Waist circumference, *HC* Hip circumference, *WHR* Waist-to-hip ratio, *WrC* Wrist circumference, *SBP* Systolic blood pressure, *DBP* Diastolic blood pressure, *TG* Triglyceride, *LDL* Low-density lipoprotein cholesterol, *HDL* High-density lipoprotein cholesterol, *FPG* Fasting plasma glucose, *OGTT* Oral glucose tolerance test, *LH* Luteinizing hormone, *FSH* Follicle stimulating hormone, *AMH* Anti-mullerian hormone, *DM* Diabetes mellitus

Data are presented as mean (SD) a, median (IQR) b, or number (%) as appropriate. ANOVA test, Kruskal–Wallis test, or χ2 test were used as appropriate

^*^Last follow up was considered a visit at which participants experienced either a DM outcome or the last follow-up which they have been censored

¥For the comparison of baseline characteristics between tertiles of serum PRL concentration; £ For the comparison of last follow up characteristics between tertiles of serum PRL concentration; 13 Statistically significant result for comparison between tertiles 1 and 3 of serum PRL concentration (*p* < 0.01); 23 Statistically significant result for comparison between tertiles 2 and 3 of serum PRL concentration (*p* < 0.01)

^†^The incidence rate of DM and the age at DM onset were not reported at baseline, as the participants with a previous diagnosis of DM were initially excluded from the study

There was no significant difference in various parameters across tertiles of PRL except for LH and LH/FSH ratio. Men with PRL within tertile three, compared separately to those with PRL within tertile two and one, had higher LH concentrations and higher LH/FSH ratios (Table 1). Point estimates and confidence intervals for the incidence rate ratio (IRR) of DM per 1000 persons in tertile 1, 2, and 3 were 2.6 (95% CI 1.9–3.7), 3.3 (95% CI 2.5–4.5), and 2.5 (95% CI 1.7–3.6), respectively. Differences in IRRs of DM between tertiles were not statistically significant (*P* = 0.15).

**Fig. 1:**
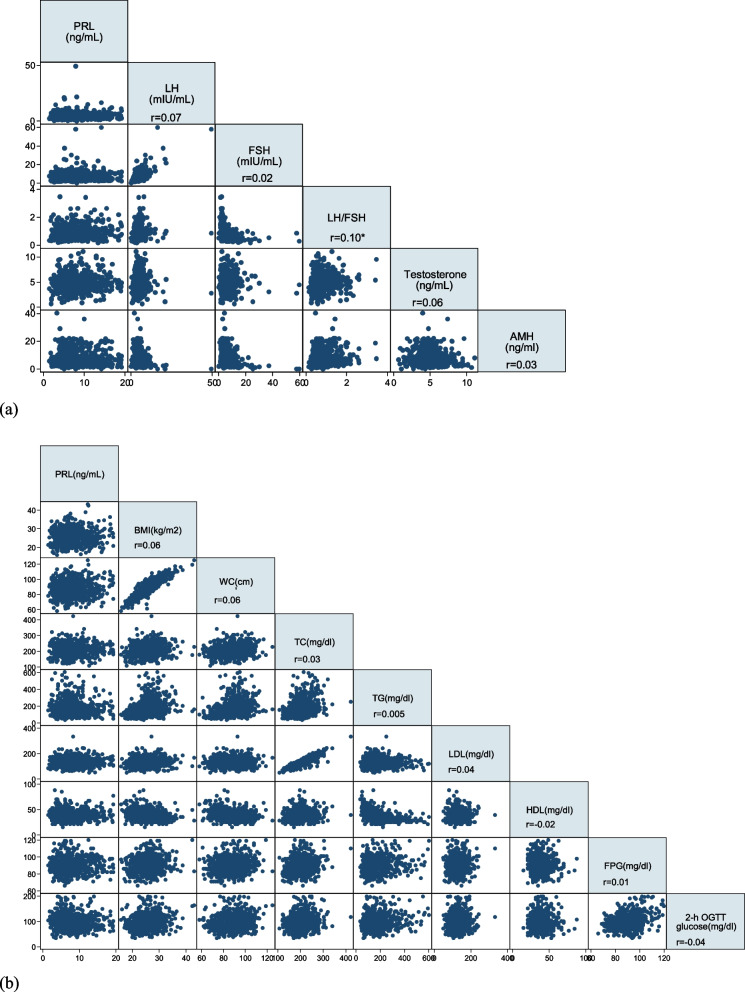
Scatter plot matrix for the association of PRL with **a** LH, FSH, LH/FSH, Testosterone, and AMH, as well as **b** indicators of glucose and lipid metabolism. Note: Baseline data were used in this analysis. The significant correlation is shown with a star (*). Abbreviations: PRL, prolactin; LH, luteinizing hormone; FSH, follicle stimulating hormone; AMH, anti-mullerian hormone

The associations between PRL and other hormones, as well as indicators of glucose and lipid metabolism, are presented based on the baseline data in Fig. 1, indicating a slightly significant positive association between PRL and LH/FSH ratio (*r* = 0.1, *P* = 0.01). However, no significant relationship was found between PRL, other hormones, and the indicators of glucose and lipid metabolism.

Adjusted and unadjusted risk ratios as well as 95% confidence intervals for type 2 DM outcome, are presented in Table 2. In the unadjusted model, PRL was not associated with the incidence rate of type 2 DM (RR = 0.98, 95% CI: 0.94 − 1.03, *P* = 0.6), while there was a significant risk reduction in type 2 DM outcome for each unit increase in LH/FSH ratio (RR = 0.55, 95% CI: 0.35 − 0.89, *P* = 0.01), testosterone (RR = 0.87, 95% CI: 0.78 − 0.98, *P* = 0.02), and AMH (RR = 0.94, 95% CI: 0.91 − 0.98, *P* = 0.01). After adjusting for age, BMI, smoking status, and family history of DM, the only significant association that remained was the inverse effect of AMH on the incidence rate of type 2 DM (RR = 0.95, 95% CI: 0.91 − 0.99, *P* = 0.02).

**Table 2:** Log-binomial model analysis (unadjusted and adjusted) for hormonal variables in relation with the incidence rate of type 2 DM

Variable	Unadjusted model		Adjusted model*	
	RR (95%CI)	*p*-value	RR (95%CI)	*p*-value
PRL (ng/ml)	0.98 (0.94,1.03)	0.6	0.97 (0.93,1.02)	0.3
LH (mIU/ml)	0.96 (0.88,1.04)	0.3	0.98 (0.89,1.07)	0.6
FSH (mIU/ml)	1.01 (0.98,1.03)	0.3	1.00 (0.97,1.04)	0.8
LH/FSH	0.55 (0.35,0.89)	**0.01**	0.76 (0.45,1.26)	0.3
Testosterone (ng/ml)	0.87 (0.78,0.98)	**0.02**	0.98 (0.88,1.11)	0.8
AMH (ng/ml)	0.94 (0.91,0.98)	**0.01**	0.95 (0.91,0.99)	**0.02**

*Abbreviations* *DM* Diabetes mellitus, *PRL* Prolactin, *LH,* Luteinizing hormone, *FSH* Follicle-stimulating hormone, *AMH* Anti-mullerian hormone

^*^Adjusted for age, BMI, smoking status, and family history of DM

Finally, an interaction term between PRL and other hormonal variables was checked in the log-binomial model, which had no significant results (not shown). The hazard ratio obtained by the Cox regression analysis for exploring the effect of PRL on the age at the onset of diabetes was close to the RRs obtained from log-binomial models (not shown).

## Discussion

The present large-scale community-based cohort study with a long follow-up found no significant association between the incidence rate of type 2 DM and serum PRL concentrations within the physiologic range among middle-aged men. Our study also indicated an inverse association between AMH, testosterone, and LH/FSH ratio with the incidence rate of type 2 DM, which except for the former, were no longer significant after adjustment for the major confounding factors, including age, BMI, smoking status, and family history of DM. In addition, the group of men with PRL within the highest tertile of the physiologic range tended to show higher concentrations of LH and LH/FSH ratio, besides the finding that PRL was weakly correlated with LH/FSH ratio.

Previous cross-sectional studies conducted in Germany and China reported that higher quartiles of PRL concentration within the physiologic range were associated with a lower prevalence of type 2 DM among both sexes [19, 20]. Another cross-sectional study on Indian individuals reported that this association was only observed in women and not in men [23]. Previous longitudinal studies that included more than 4000 American and Chinese individuals also reported that serum PRL concentrations within the physiologic range were not associated with the risk of type 2 DM among men [18, 38], which is consistent with the results of the present study. It is important to note that the population-based prospective studies investigating this association among men are scarce [22], which necessitates more studies to reach a more reliable conclusion.

PRL is associated with glucose metabolism [3, 39], which might be explained through several routes. First, PRL improves insulin sensitivity by enhancing pancreatic β cell proliferation, preventing their apoptosis, and increasing insulin secretion [5–7, 40]. Second, PRL is involved in the reproduction and fertility [30]. LH and FSH [27], testosterone [26], and AMH [28, 29] are associated with obesity, metabolic syndrome, and type 2 DM in both sexes. Moreover, the signaling of insulin receptors in the brain is suggested to be crucial to the functional integrity of the hypothalamic-pituitary–gonadal axis, implying that a link might exist between the state of insulin resistance and this axis [41]. One study by Juneja et al. conducted on male rats found that LH suppression leads to PRL rise in a chain of events, causing a surge in testosterone [42]. After a few hours, testosterone concentrations decrease, resulting in PRL suppression, followed by a surge in LH. Testosterone suppression indirectly induces the release of PRL from the pituitary. It might be inappropriate to extrapolate the findings of an animal study to humans. However, PRL and testosterone's feedback mechanism might be an evolutionarily conserved phenomenon that ensures the circadian release of PRL, which is crucial to maintaining male fertility and libido [42, 43]. Finally, PRL is involved in the regulation of lipid metabolism. Adipose tissue not only expresses PRL receptors but also secretes PRL [15, 44]. PRL reduces lipogenesis [45] and regulates the release of adiponectin and interleukin-6 (IL-6) into the adipose tissue [46, 47]. It is important to note that, although the associations between PRL, reproductive hormones, and lipid metabolism have been investigated by a few studies, it is still not clear whether the possible association between PRL and the risk of developing type 2 DM could be influenced by the regulatory effect of PRL on the other reproductive hormones, its role in the lipid metabolism, or both.

Longitudinal studies on women have found that PRL within the physiologic range is associated with the incidence rate of type 2 DM. Based on our knowledge, this was not demonstrated among men. Our prospective analyses have not indicated the abovementioned association among men, which contrasts the findings of the previous studies among women [17, 18, 38]. This issue might be due to the sex-dependent nature of the multiple mechanisms involving PRL, reproductive hormones, and adipose tissue metabolism. For instance, a sex-dependent association exists between androgens and the incidence rate of type 2 DM [48, 49]. In contrast to women, testosterone is negatively correlated with the incidence rate of type 2 DM in men. Moreover, based on the mentioned study on male rats, lower testosterone concentrations accompany higher concentrations of PRL [42]. Therefore, it can be hypothesized that lower testosterone might dilute the protective effect of higher PRL concentrations within the physiologic range on glucose metabolism among men. This might explain the lack of a prospective association between PRL concentrations within the physiologic range and the incidence rate of type 2 DM among men. On the other hand, besides the fact that PRL is involved in adipose tissue metabolism [15, 44], adipose tissue distribution is different between the sexes. For instance, visceral adipose tissue (VAT) makes up to 20% of total adipose tissue in men, compared to just 6% in women [50]. In VAT, PRL has a dose-dependent inhibitory effect on the release of IL-6. As PRL concentrations increase within the physiologic range, IL-6 release into the tissue decrease [15], leading to an inflammatory state that might aggravate insulin resistance [51]. This phenomenon is more prominent in men due to the difference in VAT volume between men and women. Another proportion of adipose tissue, called subcutaneous adipose tissues (SAT), constitutes a greater proportion of total adipose tissue in women [50]. In SAT, PRL inhibits lipolysis by down-regulating lipoprotein lipase [52], resulting in a possible favorable metabolic effect by promoting insulin sensitivity and decreasing insulin resistance [53]. In addition, adiponectin is produced in SAT, and its baseline concentration is significantly higher in women [50]. When PRL concentrations are low within the physiologic range, adiponectin production is down-regulated [54], which causes an inflammatory state that might increase insulin resistance and decrease insulin sensitivity [51]. Since women have more SAT volume than men, the metabolically favorable regulatory effect of upper-limit concentrations of PRL on lipoprotein lipase and adiponectin might be more prominent in them [51, 53, 54]. Although we are unable to verify these hypotheses due to our lack of data on women and discussed adiposity-related factors, future studies might benefit from these novel perspectives.

In the investigations regarding the role of reproductive hormones, the present study found that the incidence rate of type 2 DM in men was inversely associated with AMH concentration in both unadjusted and adjusted models. Previous research has also suggested that lower AMH concentrations in men are associated with conditions that increase the risk of type 2 DM, including metabolic syndrome and insulin resistance [28, 29]. Moreover, based on several meta-analyses, testosterone concentrations are inversely associated with the risk of developing type 2 DM in men [48, 55, 56], the findings of which were confirmed solely in the unadjusted model of the current study. This might be due to the methodologic variations of the studies included in the meta-analyses. Including studies that measured free testosterone [55], investigating only hypogonadal men [56], using a limited number and relatively modest size of studies included [56], and lack of adjustment for confounders such as the family history of DM and smoking status [48, 55, 56] are the major aspects that might partly explain the contradiction between their results and ours. However, in a recent cohort study conducted on a sample of 673 middle-aged Chinese men, Li et al. [57] reported that in an adjusted model that also included smoking status, the odds of incident type 2 DM were not increased in different quartiles of total testosterone. The authors also reported the same results for LH and FSH, which agrees with our findings. However, an inverse association between the incidence rate of type 2 DM and concentrations of LH and FSH in males has been suggested by other studies [58, 59]. In addition, testosterone which is negatively associated with the risk of developing type 2 DM, is positively associated with LH and FSH concentrations in men [60], further contributing to the idea. Another study was conducted on knockout mice lacking neural insulin receptors to mimic the state of insulin resistance and found a significant reduction in LH concentrations in both sexes [41]. The authors concluded that the means to maintain the normal function of the hypothalamic-pituitary–gonadal axis is through regulating LH concentration. This indicates that type 2 DM might be associated with a lower LH/FSH ratio, explaining the significant inverse association between the incidence rate of type 2 DM and the LH/FSH ratio found in the unadjusted model of the present study. In addition, the LH/FSH ratio was positively correlated with PRL concentrations within the physiologic range in our study. This correlation might be justified by the observation made in the experimental study by Juneja et al., in which the stimulation of PRL pituitary release is preceded by a surge in LH among male rats [42]. Of note, the association between LH and higher PRL concentration which suggests a decrease in dopaminergic tone was also reported in our recent study [61].

This study has benefited from a prospective community-based design with a large sample of middle-aged men followed for more than 15 years. Another strength of this study arises from investigating the association between PRL and other reproductive hormones possibly involved in the mechanisms of glucose metabolism among men, including LH, FSH, testosterone, and AMH, which was lacking in the literature. There are also some limitations in this study worth mentioning. First, for women of the Tehran Lipid and Glucose Study, we lacked the required data to explore the associations between PRL, type 2 DM, and hormonal variables. Such data would enable us to compare these associations between the sexes with evidential accuracy. Second, a single measurement of PRL concentration was performed for each participant, which may not be the most reliable option, considering it is affected by factors like physical activity and stress. However, based on the guidelines for population-based studies, a single measurement is usually enough if the aim is overall assessment [62]. In addition, all proceedings were taken into account to make that single measurement as accurate as possible, which is mentioned in the methods section. Third, macroprolactin screening was not performed in asymptomatic men with serum PRL concentrations above the physiologic range. However, it should be noted that most current guidelines recommend a case-finding approach rather than a systematic screening for macroprolactinemia [63]. Fourth, testosterone was measured in the form of total testosterone (TT), consisting of bound plus free testosterone (FT). FT, as the bioavailable form of testosterone, yields more accurate results compared to TT. However, due to its standard equilibrium dialysis method’s complexity and its unharmonized assays, measuring FT is challenging in clinical laboratories [57]. In addition, faulty models of the bond between testosterone and sex-hormone binding globulin were used to derive FT calculating algorithms [64]. Finally, the markers of adiposity were not measured in this study, which could further shed light on the sex-specific association between PRL and t the incidence rate of type 2 DM. To better understand the mechanism underlying the effect of PRL on metabolism in various situations, further comprehensive studies, including assessment of different influential factors and adiposity factors with long enough follow-up, are recommended.

## Conclusions

In a large-scale community-based cohort study, no significant association was found between serum prolactin concentrations within the physiologic range and the incidence rate of type 2 diabetes mellitus among middle-aged men. There was a tendency for men with higher concentrations of PRL within the physiologic range to show higher levels of LH and LH/FSH. These factors, along with FSH and testosterone, do not appear to be linked to type 2 DM in men. Higher AMH, however, was linked to a lower risk of developing type 2 DM in them.

## Data Availability

The datasets generated during and analyzed during the current study are not publicly available due to it being part of a large-scale cohort study called the Tehran Lipid and Glucose Study (TLGS), but are available from the corresponding author on reasonable request.

## Abbreviations

PRL: Prolactin
DM: Diabetes Mellitus
LH: Luteinizing hormone
FSH: Follicle-stimulating hormone
AMH: Anti-mullerian hormone
NCDs: Non-communicable diseases
WC: Waist circumference
HC: Hip circumference
WrC: Wrist circumference
BMI: Body mass index
WHR: Waist-to-hip ratio
FPG: Fasting plasma glucose
TC: Total cholesterol
TG: Triglyceride
HDL: High-density lipoprotein
LDL: Low-density lipoprotein
IL-6: Interleukin-6
VAT: Visceral adipose tissue
SAT: Subcutaneous adipose tissue
FT: Free testosterone
